# Assessing short-term impact of PM_10_ on mortality using a semiparametric generalized propensity score approach

**DOI:** 10.1186/s12940-020-00599-6

**Published:** 2020-05-01

**Authors:** Laura Forastiere, Michele Carugno, Michela Baccini

**Affiliations:** 1 Department of Statistics, Computer Science, Applications, University of Florence, Viale Morgagni 59, Florence, 50134 Italy; 2 Department of Biostatistics, Yale School of Public Health, New Haven, CT US; 3 Department of Clinical Sciences and Community Health, University of Milan and Fondazione IRCCS Ca’ Granda Ospedale Maggiore Policlinico, Milan, Italy

**Keywords:** Short-term effects of air pollution, Health impact assessment, Attributable deaths, Generalized propensity score, Potential outcomes, Exposure-response function

## Abstract

**Background:**

The shape of the exposure-response curve describing the effects of air pollution on population health has crucial regulatory implications, and it is important in assessing causal impacts of hypothetical policies of air pollution reduction.

**Methods:**

After having reformulated the problem of assessing the short-term impact of air pollution on health within the potential outcome approach to causal inference, we developed a method based on the generalized propensity score (GPS) to estimate the average dose-response function (aDRF) and quantify attributable deaths under different counterfactual scenarios of air pollution reduction. We applied the proposed approach to assess the impact of airborne particles with a diameter less than or equal to 10 *μ*m (PM_10_) on deaths from natural, cardiovascular and respiratory causes in the city of Milan, Italy (2003-2006).

**Results:**

As opposed to what is commonly assumed, the estimated aDRFs were not linear, being steeper for low-moderate values of exposure. In the case of natural mortality, the curve became flatter for higher levels; this behavior was less pronounced for cause-specific mortality. The effect was larger in days characterized by higher temperature. According to the curves, we estimated that a hypothetical intervention able to set the daily exposure levels exceeding 40 *μ*g/m^3^ to exactly 40 would have avoided 1157 deaths (90%CI: 689, 1645) in the whole study period, 312 of which for respiratory causes and 771 for cardiovascular causes. These impacts were higher than those obtained previously from regression-based methods.

**Conclusion:**

This novel method based on the GPS allowed estimating the average dose-response function and calculating attributable deaths, without requiring strong assumptions about the shape of the relationship. Its potential as a tool for investigating effect modification by temperature and its use in other environmental epidemiology contexts deserve further investigation.

## Background

In investigating short-term effects and impacts of air pollution on population mortality and morbidity, the exposure-response relationship is frequently assumed to be linear on a logarithmic scale, supporting the idea that an increase of the air pollution level yields the same percent increase in the occurrence of health events at any exposure level. This choice is in line with part of the epidemiological literature which does not report evidences of strong deviations from log-linearity within the ranges of exposure levels observed in urban areas [1–10]. However, recent works suggest a possible violation of the log-linearity assumption, especially in contexts where the range of exposure values was sufficiently wide to allow exploring the curve also for high air pollutant levels. For example, in a study aimed at investigating the relationship between the concentration of atmospheric particulate matter having diameter less than or equal to 2.5 *μ*m (PM _2.5_) and mortality in 272 Chinese cities, a leveling off in the exposure-response curves at high concentrations was reported in some areas of the Country [11]. Another Chinese study found a non log-linear increase in the occurrence of respiratory diseases for daily PM _2.5_ levels above 50 *μ*g/m^3^ [12]. Evidences of violation of the log-linearity assumption were found also in Li et al. [13] for the relationship between PM _2.5_ and mortality and in Zu et al. [14] for the relationship between ozone and hospitalizations from asthma. Finally, in the recent study conducted by Liu and colleagues on 652 cities around the world, the estimated pooled exposure-response functions between daily average levels of PM_10_ and PM _2.5_ and all-cause mortality showed no thresholds and seemed to flatten at high air pollutants concentrations [15].

The shape of the exposure-response relationship has important regulatory implications. As a matter of fact, in the absence of a safe threshold below which exposure has no effect on mortality (which is the case of a log-linear exposure-response relationship), any reduction of the air pollutant concentrations is expected to bring to a reduction of the mortality/morbidity burden attributable to the exposure, even when air pollution levels are already under the actual regulatory limits: as such, this suggests the opportunity of policies even in areas characterized by low-moderate levels of air pollution. On the other hand, should there be a safe threshold, regulations reducing concentrations already below such a threshold would be considered to have a small impact on public health [10]. This is also why the shape of the exposure-response relationship, as related to the existence of a safe threshold for air pollutant concentration, has been a matter of debate both within the scientific community and across regulatory agencies [16, 17].

A second aspect related to log-linearity concerns the quantification of mortality and disease burdens in the population. In fact, it was shown that even in case of mild violation of log-linearity, assuming log-linearity could lead to under- or over-estimate the impact of air pollution, intended as number of health events attributable to the exposure [6]. Despite this, it is still common practice to estimate impacts assuming a log-linear effect of the exposure (see for example [15]). Gasparrini and Leone [18] did propose a method to estimate the fraction of health events attributable to extreme ambient temperatures from a flexible exposure-response curve. However, their approach, although potentially applicable to assess also the short term impact of air pollution, is not yet common practice in this field.

Up to now, the studies that explored the violation of the log-linearity assumption in the context of the short-term effects of air pollution usually described the exposure-response function by including flexible terms in the Poisson or quasi-Poisson regression model specified on the daily number of health events, for example a regression spline or a penalized spline defined on the daily air pollutant concentrations [7, 12, 14, 15, 19, 20]. Alternative approaches to regression have never been adopted.

The potential outcomes approach to causal inference [21, 22] was recently considered a useful tool in the policy debate about air pollution regulatory interventions [23], as it encourages to think in terms of causes and effects, within a formal mathematical framework. The causal interpretation of the estimated associations is particularly relevant when the effect estimates are used to calculate the absolute mortality and disease burden related to the air pollution exposure. Some studies adopted causal inference methods in the analysis of short-term effects of air pollution on population health, but none of these explored the shape of the exposure-response function [24, 25].

The generalized propensity score (GPS) method allows a flexible modeling of the exposure-response function within a potential outcomes approach to causal inference. The GPS was introduced by Hirano and Imbens [26] as a generalization of the propensity score (PS) – used in the case of a binary treatment – to the case of discrete treatments, continuous treatments and arbitrary treatment regimes [27–30]. As opposed to standard regression adjustment, the GPS-based adjustment allows adjusting for the potentially non-linear effect of a large number of covariates and it results in a flexible way to explore effect modification with every characteristic.

In the present paper, we proposed to use a semiparametric GPS method for covariate adjustment to estimate the exposure-response function describing the short-term effect of the air pollutant exposure on the health outcome, in the context of epidemiological time series analyses. In particular, we built on the semiparametric GPS proposed by Bia and colleagues [31], replacing the normal model for the outcome with a quasi-Poisson model, more suitable for count data, and including spline terms to account for non-linearities. In addition, we implemented a novel procedure for the estimation of the attributable number of events according to the estimated exposure-response curve, assuming different counterfactual scenarios.

In this paper, we also defined ad hoc estimands to investigate the role of temperature in modifying the effect of air pollution. As a matter of facts, a major point in the study of the short-term effects of air pollution on health is the possible synergic effect between exposure to air pollution and ambient temperature. This issue is particularly relevant if we consider that, according to the climate change projections, number and intensity of extreme weather days are expected to increase in the future [32]. In the literature, there is some evidence that the association between air pollution and mortality is stronger during summer or in days with high temperatures [33–36], but further investigation is needed.

We applied the proposed approach to the estimation of short-term effects and impacts of particulate matter with a diameter less than or equal to 10 *μ*m (PM_10_) on mortality from all natural, respiratory, and cardiovascular causes in the city of Milan, Italy for the period 2003-2006. This data set has already been analyzed according to the standard method based on Poisson regression [37, 38] and, more recently, using propensity score-based matching with a dichotomous version of the exposure (high levels versus low levels of PM_10_) [25]. This allowed us to compare our results to the findings of these previous studies.

## Methods

### Data

We considered data for the city of Milan (1,299,633 inhabitants in 2007) for the years 2003-2006. Milan is located in an area where unfavorable geographical and climate conditions induce frequent phenomena of thermal inversion, exacerbating the air pollution mainly due to traffic and, to a minor extent, to non-industrial combustion [39]. The daily time series of PM_10_ levels, temperature and humidity were obtained by averaging data over the available air quality monitoring stations of the Regional Environmental Protection Agency (ARPA Lombardia). Daily mortality data were obtained from the Regional Mortality Register. We focused on the deaths of the resident population occurred within the city area. We considered daily mortality from all natural (International Classification of Diseases, Ninth Revision (ICD-9) codes below 800), cardiovascular (ICD-9: 390-459) and respiratory (ICD-9: 460-519) causes.

### Notation and potential outcomes

Let *i*=1,…,*N* be the indicator of the day, which will be also referred to as the unit. Let $Z_{i} \in \mathcal {Z}$ be the exposure level in day *i*, defined as the average level of PM_10_ in the current day *i* and in the previous one *i*−1 (lag 0-1 exposure), and let $Y_{i} \in \mathcal {Y}$ be the number of deaths in day *i*. Finally, let $\mathbf {X}_{i} \in \mathcal {X}$ be a vector of *K* covariates for day *i*, which includes meteorological variables (ambient temperature and humidity), seasonality terms, holidays and influenza epidemics indicators.

According to the potential outcome framework [21, 22], we denote by *Y*_*i*_(*z*) the potential number of deaths in day *i* if *z* were the exposure level in that day. For each day a collection of potential outcomes is defined, one for each possible level of exposure *z*, but we only observe the one corresponding to the actual exposure of that day, *Z*_*i*_, being *Y*_*i*_(*Z*_*i*_)=*Y*_*i*_. Thus, our effort is to define a procedure that allows us to extrapolate information on the unobserved potential outcomes across days with similar baseline covariates.

Note that potential outcomes of the form *Y*_*i*_(*z*), with $z \in \mathcal {Z}$ are well-defined only under the Stable Unit Treatment Value Assumption (SUTVA) [22], which rules out the presence of interference between units, i.e., the possibility that the potential outcomes of one day are affected by the level of exposure in previous days. In this paper, we considered the lag 0-1 exposure not only to allow comparison with previous results, but also to prevent interference across days as required by SUTVA. In fact, using the lag 0-1 exposure instead of the current PM_10_ level makes the no-interference assumption more plausible [25]. Enlarging the window of the moving average for the exposure definition would have made the no-interference assumption even more plausible, but at the price of a lower variability of the exposures and of a reduced possibility of detecting an impact.

### Causal estimands

In our analysis we focused on different causal estimands. We defined the average dose-response function (aDRF) as:

1 $$ \mu(z)=\frac{1}{N}\sum_{i}Y_{i}(z),  $$

which represents the average daily number of deaths we would have observed fixing the exposure to *z* in each day during the study period. We estimated this quantity precisely to capture the average causal relationship between the exposure level and mortality rate in the city of Milan during the years 2003-2006. We also defined conditional average dose-response functions of the type:

2 $$ \mu(z; \mathbf{x})=\frac{1}{N(\mathcal{X}^{\star})}\sum_{i:\mathbf{X}_{i} \in \mathcal{X}^{\star}}Y_{i}(z),  $$

where $N(\mathcal {X}^{\star })$ is the number of days with covariate levels included in the subset $\mathcal {X}^{\star }$ of $\mathcal {X}$. We used this kind of estimand to investigate how the relationship between exposure and outcome changed according to the levels of air temperature.

In addition to the average dose-response functions, we also focused on the absolute number of deaths attributable to observed levels of exposure exceeding a pre-specified counterfactual threshold *z*^⋆^. In the epidemiological literature, this quantity is usually referred to as *attributable deaths (AD)* and can be expressed as:

3 $$  AD(z^{\star})=\sum_{i: Z_{i}>z^{\star}} (Y_{i} - Y_{i}(z^{\star})).  $$

In other words, *AD*(*z*^⋆^) compares the observed mortality in days with *Z*_*i*_>*z*^⋆^ to the mortality that we would have observed had the exposure been set exactly equal to *z*^⋆^.

As an alternative to *AD*(*z*^⋆^), we quantified the number of attributable deaths with reference to a more complex, but realistic, counterfactual scenario, reflecting a hypothetical intervention that, instead of fixing the exposure to a specific value *z*^⋆^, would replace the observed exposures when *Z*_*i*_>*z*^⋆^ with exposure values drawn from an observed or hypothetical distribution *p*^⋆^(*z*). This new causal estimand, referred to as *distributional attributable deaths (DAD)*, takes the following form:

4 $$  {DAD(p^{\star}(z),z^{\star})=\int_{z} \left(\sum_{i: Z_{i}>z^{\star}} \left(Y_{i} - Y_{i}(z)\right)\right)p^{\star}(z)dz.}  $$

In our analysis, we defined *p*^⋆^(*z*) as the actual distribution of the exposure in the days where *Z*_*i*_≤*z*^⋆^, that is, *p*^⋆^(*z*)=*p*(*z*|*Z*_*i*_≤*z*^⋆^). For simplicity, we denoted the corresponding estimand by *DAD*(*z*^⋆^).

Under the assumption that all relevant confounders have been measured, also known as the unconfoundedness assumption or the no-unmeasured confounders assumption^1^, the aDRF and the other estimands presented in the previous section were identifiable and could be estimated without bias conditioning on covariates. In our analysis, the validity of the unconfoundedness assumption, which cannot be tested on data, relied on the fact that the selection of the covariates **X**_*i*_ was based on a substantive a priori knowledge of the phenomenon derived from the literature on the analysis of short term effects of air pollution, from the seminal papers [40–42] to the very recent work by Liu et al. [15].

### Generalized propensity score

According to Hirano and Imbens [26], the GPS, denoted by *r*(*z*;**x**), is defined as the conditional density of the exposure *Z* given the covariates **X**:

5 $$  r(z; \mathbf{x})=p_{Z|\small\mathbf{X}}(z|\mathbf{x}).  $$

Similarly to the PS, the GPS is a balancing score, meaning that within strata of units with the same value of *r*(*z*;**x**), the probability that *Z*_*i*_ is equal to *z* does not depend on the covariates value [43]. The unconfoundedness assumption, coupled with the balancing property of the GPS, implies that assignment to treatment is unconfounded given the GPS^2^. In practice, this means that any bias given by differences in the distribution of covariates across groups with different exposure levels can be removed by adjusting for the GPS [26].

In this paper, we used the GPS to obtain estimates of the causal estimands described in the previous section. For this purpose, first we parametrically modeled and estimated the GPS through a model for the exposure, then we specified and estimated a model for the outcome given the GPS and the exposure, and we used it to obtain estimates of the causal quantities of interest.

#### Model for the exposure

We assumed a parametric log-normal model for the exposure *Z*_*i*_:

6 $$  \log\left(Z_{i}\right)\sim N\left(\alpha_{0} +\vec{\alpha}_{X}^{T}\mathbf{X}_{i}, \sigma_{Z}\right),  $$

where *α*_0_ and $\vec {\alpha }_{X}$ were unknown regression coefficients and *σ*_*Z*_ was the unknown variance. In particular, we included in the model the following regressors: indicators of day of week, holiday and influenza epidemics, a linear regression spline with 5 degrees of freedom per year and equally spaced knots on the calendar day to account for seasonality and long-term trend, a cubic regression spline for temperature at lag 0-3 with 5 degrees of freedom and knots at the quantiles, linear and quadratic terms for relative humidity, and an indicator of the July-August period to account for the reduction of the population present in the city during the summer season [15, 37].

According to model 6, the GPS for day *i* at the level of exposure *z* was thus defined as:

7 $$ r\left(z;\mathbf{X}_{i}\right)=\frac{1}{\sqrt{2\pi\sigma_{Z}^{2}}}\exp{-\frac{\left(\log\left(z\right)-\alpha_{0}-\vec\alpha_{X}^{T}\mathbf{X}_{i}\right)^{2}}{2\sigma_{Z}^{2}}}.  $$

In the same way, we defined the actual GPS for day *i*, i.e. the GPS evaluated at the level of exposure actually observed, as:

8 $$ {}R_{i}=r\left(Z_{i};\mathbf{X}_{i}\right)=\frac{1}{\sqrt{2\pi\sigma_{Z}^{2}}}\exp{-\frac{\left(\log\left(Z_{i}\right)-\alpha_{0}-\vec\alpha_{X}^{T}\mathbf{X}_{i}\right)^{2}}{2\sigma_{Z}^{2}}}.  $$

It is worth noticing that different specifications were possible for the model 6 and some effort was needed to find the most appropriate one. A key criterion driving the specification of the model consists in checking the balancing property of the estimated GPS. In order to check the balancing property of the GPS, we applied the method described in Hirano and Imbens [26]. After creating four classes of exposure, *C*_1_=(0,20),*C*_2_=[20,40),*C*_3_=[40,70),*C*_4_=[70+), we applied the following procedure for each covariate *X*_*i*_. First, we checked the independence of each covariate and each exposure class indicator 1{*Z*_*i*_∈*C*_*k*_} by calculating the marginal *t* statistics for the mean difference *X*_*i*_ between the class *C*_*k*_ and all the others as a whole. Then, we calculated for each day the GPS at the median of each treatment class (*M*_*k*_,*k*=1,…,4) and we checked the independence of each covariate *X*_*i*_ and each exposure class indicator 1{*Z*_*i*_∈*C*_*k*_}, conditional on *r*(*M*_*k*_;***X***_*i*_). In order to do this, given the class *C*_*k*_, we defined four blocks according to the quartiles of *r*(*M*_*k*_,***X***_*i*_) and, for each block, we calculated the mean difference of *X*_*i*_ between days belonging to the class *C*_*k*_ and days belonging to the other three classes, as well as the corresponding standard error. The four differences obtained, one for each GPS block, were then combined in a weighted mean, with weights proportional to the number of observations in each block. A *t* statistics was thus calculated as the ratio of the combined difference and its standard error. From the descriptive comparison between these GPS-adjusted *t* statistics and marginal *t* statistics (four comparisons for each variable, one for each class *C*_*k*_), we were able to evaluate if adjusting for GPS improved the balance.

#### Model for the outcome

We specified a quasi-Poisson model for the outcome given the exposure and the GPS:

9 $$  Y_{i}(z) \sim \textrm{quasi-Poisson}(\lambda_{i}) \quad \log(\lambda_{i})=s\left(z, r(z;\mathbf{X}_{i})\right),  $$

where *s*(*z*,*r*(*z*;**X**_*i*_)) was a penalized bivariate splines, with radial basis functions defined on a large number of knots^3^. The smoothing parameter was automatically selected by Generalized Cross Validation [44].

### Estimation procedure

The estimation procedure consisted in the following steps:

1. We estimated the parameters of the exposure model, $\hat {\alpha }_{0}, \hat {\vec \alpha }_{X}$ and $\hat {\sigma }^{2}_{Z}$ ;
2. We used them to predict for each day *i* the actual GPS, $\widehat {R}_{i}$^4^ ;
3. We used the observed data (*Y*_*i*_,*Z*_*i*_) and $\widehat {R}_{i}$ to estimate the outcome model;
4. For each exposure level *z* and for each unit *i*, we predicted the potential outcome *Y*_*i*_(*z*) using the following steps:
  a We predicted the GPS at that level of exposure *z*, $\hat {r}(z; \mathbf {X}_{i})$^5^ ;
  b We used the estimated outcome model and $\hat {r}(z; \mathbf {X}_{i})$ to predict the potential outcome at *z*, $\widehat {Y}_{i}(z)$.
5. We used the predicted potential outcomes to estimate the quantities of interest. In particular, we estimated the average and the conditional dose-response functions by averaging the potential outcomes over all units
  $$\widehat{\mu}(z)=\frac{1}{N} \sum_{i=1}^{N}\widehat{Y}_{i}(z)$$
  and over all units with **X**_*i*_ included in each subset $\mathcal {X}^{\star }$ of interest
  $$\widehat{\mu}(z, \mathbf{x})=\frac{1}{N(\mathcal{X}^{\star})} \sum_{i: \mathbf{X}_{i}\in \mathcal{X}^{\star}}\widehat{Y}_{i}(z)$$
  While *AD*(*z*^⋆^) was estimated by the following sum over all units with *Z*_*i*_>*z*^⋆^
  $$\widehat{AD}(z^{\star})=\sum_{i: Z_{i}>z^{\star}} Y_{i} - \widehat{Y}_{i}(z^{\star}),$$
  a more complex procedure was needed for *DAD*(*z*^⋆^). For the estimation of *DAD*(*z*^⋆^) we repeated the following steps M times:
  a We drew a value *z*^*m*^ from the distribution *p*^⋆^(*z*)
  b We computed
    $$\widehat{AD}^{m}=\sum_{i: Z_{i}>z^{\star}} \left(Y_{i} - \widehat{Y}_{i}\left(z^{m}\right)\right)$$
  and we finally calculated the average $\widehat {DAD}(z^{\star })=\frac {1}{M}\sum _{m}^{M} \widehat {AD}^{m}$.

Standard errors and 90% confidence intervals of the causal estimands were estimated with a bootstrap method [26, 45]. Bootstrap samples were obtained by using an independent resampling strategy with replacement. For each bootstrap sample the parameters of the exposure and outcome models were estimated and causal estimands were computed using the above estimation procedure.

## Results

Summary statistics of daily counts of deaths, PM_10_ concentrations and temperature levels are reported in Table 1.

**Table 1:** Summary statistics for daily counts of deaths, PM_10_ concentrations and temperatures, Milan (2003-2006)

Variable	Mean	Std. Dev.	Median	Min	90^*th*^ pct	Max
Natural Deaths	28.0	7.0	28	9	37	69
Cardiovascular Deaths	10.3	3.9	10	0	15	27
Respiratory Deaths	2.5	1.7	2	0	5	11
PM_10_ (*μ*g/m^3^)	52.5	32.9	43.5	3.5	99.5	227.1
Temperature (^∘^C)	14.5	8.4	14.3	-2.0	26.0	31.8

In order to check the balancing property of the GPS we focused on the following variables: average daily temperature at lag 0-3, extreme temperature indicator (days with average temperature at lag 0-3 exceeding the 95^*th*^ percentile calculated over the study period), humidity at lag 0, and indicators for season (summer/winter), weekends, holidays and influenza epidemics. We found that, after the adjustment for the GPS calculated according to the model specification described in the *Model for the exposure* section, the *t* statistics comparing the average of each covariate between classes of exposure reduced in respect to the marginal *t* statistics, in particular when the initial imbalance between groups was strong (Table 2). This was indicative that the balancing property was satisfied. More complex models for GPS, involving splines with a larger number of knots, a spline for humidity and interactions terms, did not bring to further improvement of the balance (results not reported).

**Table 2:** Results of the balancing check for the generalized propensity score (GPS)

Covariate	Class of exposure	Marginal *t*	GPS-adjusted *t*
Temperature (lag 0-3)	(0, 20)	5.60	-2.57
	[20, 40)	14.15	1.68
	[40, 70)	-0.39	-0.26
	[70+)	-20.78	-3.00
Influenza epidemics	(0, 20)	-2.80	-0.44
	[20, 40)	-7.47	-2.87
	[40, 70)	1.32	3.43
	[70+)	8.92	0.47
Summer indicator	(0, 20)	7.93	-2.19
	[20, 40)	14.30	2.39
	[40, 70)	-3.09	-0.77
	[70+)	-18.46	-2.48
Holidays indicator	(0, 20)	1.91	-0.07
	[20, 40)	0.02	-0.05
	[40, 70)	-2.74	-2.81
	[70+)	1.97	2.58
Humidity (lag 0)	(0, 20)	-5.78	-0.13
	[20, 40)	-5.91	1.05
	[40, 70)	-0.15	-2.78
	[70+)	10.69	1.78
Weekend indicator	(0, 20)	4.92	2.27
	[20, 40)	1.79	-1.33
	[40, 70)	-1.39	-1.13
	[70+)	-3.35	0.64
Heat episodes	(0, 20)	-2.25	-0.96
	[20, 40)	1.77	-0.15
	[40, 70)	3.48	2.43
	[70+)	-4.73	-1.99

Marginal and GPS-adjusted *t* statistics for the mean difference between one class of exposure and the others as a whole, for a set of selected covariates

**Fig. 1:**
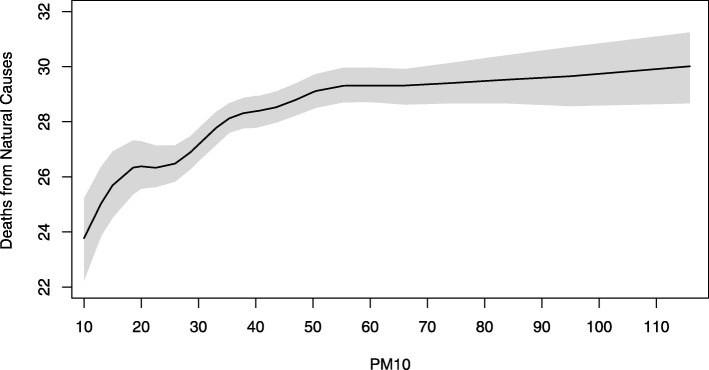
Average Dose-Response Function for natural mortality. Average dose-response function (90% pointwise confidence band) of the causal relationship between PM_10_ exposure at lag 0-1 and average daily mortality from natural causes

**Fig. 2:**
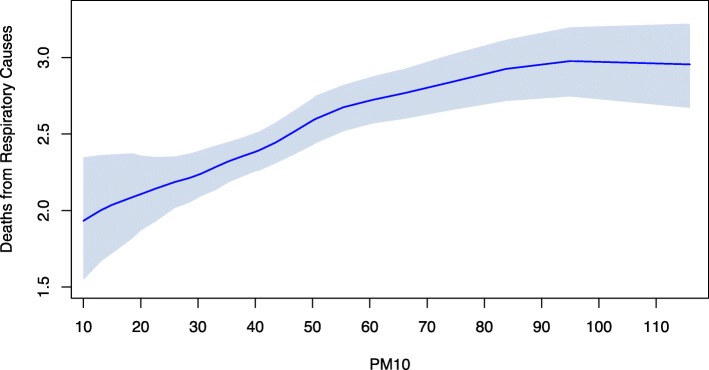
Average Dose-Response Function for respiratory mortality. Average Dose-Response Function (90% pointwise confidence band) of the causal relationship between PM_10_ exposure at lag 0-1 and average daily mortality from respiratory causes

**Fig. 3:**
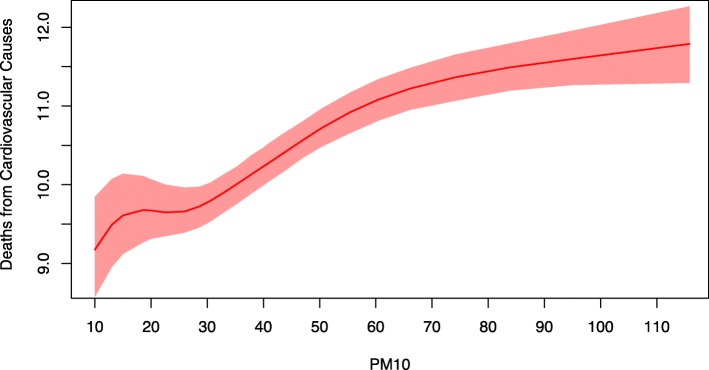
Average Dose-Response Function for cardiovascular mortality. Average Dose-Response Function (90% pointwise confidence band) of the causal relationship between PM_10_ exposure at lag 0-1 and average daily mortality from cardiovascular causes

In Fig. 1 we report the estimated aDRF for natural-cause mortality, which describes how the average level of mortality, defined as in equation 1, changes according to the PM_10_ level at lag 0-1. The relationship was increasing and nonlinear, steeper at low concentrations and flat above 50-60 *μ*g/m^3^. The aDRFs estimated for respiratory and cardiovascular mortality had a similar shape (Figs. 2 and 3). However, the tendency of these curves to flatten was less pronounced and, when present, arose at even higher PM_10_ concentrations.

Especially for natural-cause and cardiovascular mortality, the shape of the relationship was unexpectedly wiggly at low levels of exposure. This was probably due to an excessive local dependence of the curve on the knots location of the bivariate spline in equation 9, related to the fact that information under 20 *μ*g/m^3^ was quite poor, relying on only 6% of days.

**Fig. 4:**
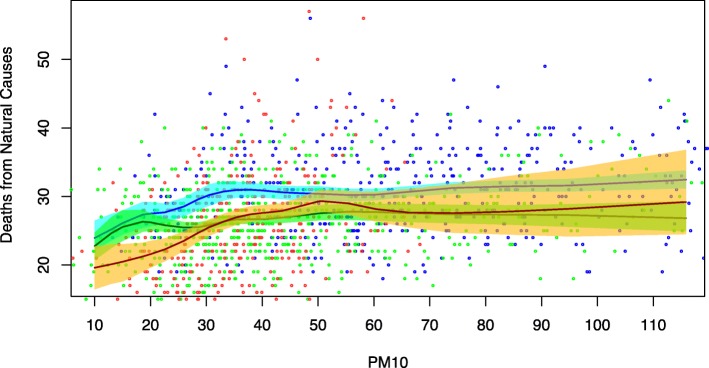
Average Dose-Response Function for natural mortality by temperature level. Scatterplot and average Dose-Response Functions (90% pointwise confidence bands) of the causal relationship between PM_10_ exposure at lag 0-1 and average daily mortality from natural causes, by level of temperature at lag 0-3: up to 10^∘^C (blue), 10/23^∘^C (green), over 23^∘^C (red)

For natural-cause mortality, we also estimated the aDRF within three different categories of days, defined according to daily average temperature levels (at lag 0-3): low (up to 10^∘^C), medium (10/23^∘^C), high (over 23^∘^C) (Fig. 4). As expected, background mortality was higher in days characterized by lower temperatures (blue dots) and lower in days with higher temperatures (red dots); however, the slope of the exposure-response association appeared to be slightly steeper in days belonging to the higher temperature category, suggesting a possible synergic effect between PM_10_ exposure and heat.

After estimating the aDRFs, we estimated the causal estimands introduced in the *Causal estimands* section. For the three outcomes of interest, we estimated *AD*(20),*AD*(40),*DAD*(40) and *DAD*(50) (Table 3). The choice of the threshold *z*^⋆^ of the countefactual scenarios followed internationally recognized standards, in details:

- 20 *μ*g/m^3^ is the World Health Orgaization (WHO) Air Quality Guideline threshold [46] for PM_10_ annual average;

**Table 3:** Attributable deaths in Milan for natural, respiratory and cardiovascular causes from 2003 to 2006: estimates and 90% confidence intervals calculated according to different counterfactual scenarios of PM_10_ reduction

Estimand	Natural Deaths		Respiratory Deaths		Cardiovascular Deaths	
	Estimate	90% CI	Estimate	90% CI	Estimate	90% CI
*AD*(20)	2537	(1273, 3655)	577	(226, 900)	1000	(464, 1488)
*AD*(40)	1157	(689, 1645)	312	(210, 418)	771	(580, 961)
*DAD*(40)	1857	(1479, 2233)	403	(293, 504)	1014	(815, 1221)
*DAD*(50)	1481	(1212, 1735)	325	(251, 398)	828	(690, 960)

*AD*(20): deaths attributable to daily exposure levels above 20 *μ*g/m^3^, setting the counterfactual exactly to 20 *μ*g/m^3^.

*AD*(40): deaths attributable to daily exposure levels above 40 *μ*g/m^3^, setting the counterfactual exactly to 40 *μ*g/m^3^.

*DAD*(40): deaths attributable to daily exposure levels above 40 *μ*g/m^3^, sampling the counterfactuals from the exposure distribution below 40 *μ*g/m^3^.

DAD(50): deaths attributable to daily exposure levels above 50 *μ*g/m^3^, sampling the counterfactuals from the exposure distribution below 50 *μ*g/m^3^
- 40 *μ*g/m^3^ is the European Union (EU) limit for PM_10_ annual average [47];
- 50 *μ*g/m^3^ is the EU limit for PM_10_ daily average, not to be exceeded for more than 35 days per year [47].

Exceeding the threshold of 20 *μ*g/m^3^ has been responsible of the largest impact. In particular, an hypothetical intervention able to set the air pollution level in all days with exposure above 20 *μ*g/m^3^ to exactly 20 *μ*g/m^3^ (*AD*(20)), would have avoided 2537 deaths (90% Confidence Interval (CI): 1273, 3655), 577 (90% CI: 226, 900) of which from respiratory causes and 1000 (90% CI: 464, 1488) from cardiovascular diseases. The lowest impact was estimated for an intervention able to set PM_10_ concentration levels higher than 40 *μ*g/m^3^ to exactly 40 *μ*g/m^3^ (*AD*(40)): 1157 (90% CI: 689, 1645) attributable deaths, 312 (90% CI: 210, 418) for respiratory causes and 771 (90% CI: 580, 961) for cardiovascular causes. The fact that attributable deaths for cardiovascular and respiratory causes represented a variable proportion of the total impact was directly related to the shape of the exposure-response functions.

With $DAD(p^{\star }_{40}$), we quantified the impact of a hypothetical intervention able to set the air pollution levels in all days with exposure above 40 *μ*g/m^3^ to values belonging to the PM_10_ distribution observed in days with exposure below 40 *μ*g/m^3^. As expected, *DAD*(40) was larger than *AD*(40), because it also picked counterfactual levels of the exposure far below the chosen threshold of 40 *μ*g/m^3^. For the same reason, also *DAD*(50), with almost 1500 attributable deaths, returned an impact larger than *AD*(40), despite the chosen threshold was higher. In both *DAD* scenarios, attributable cardiovascular deaths were about 55% of all natural attributable deaths, while respiratory mortality represented about 22% of the total.

## Discussion

In this paper, we investigated the shape of the exposure-response function describing the short-term effect of PM_10_ on mortality, by using a semi-parametric procedure based on the GPS [31]. To the best of our knowledge, this is the first time that the GPS approach is applied in the analysis of short-term effects of air pollution. A recent work by Wu and colleagues [48] did propose a somewhat similar approach, but applied it to the analysis of long-term effects of air pollution; in addition, they did not quantify the mortality burden of air pollution in terms of attributable health events. On the contrary, we developed a novel procedure for the estimation of the attributable deaths based on the estimated curve, considering alternative counterfactual scenarios.

As opposed to regression-based methods, including those based on splines (e.g., Gasparrini and Leone [18]), the GPS-based method we propose allows adjusting for all possible covariates including non-linearities and interactions with the exposure - the same being more difficult in a regression model - and is less prone to incur into issues of overfitting, multicollinearity and variance inflation.

Attributable events calculation within a causal inference framework is not new in the analysis of the short-term effects of air pollution on health. In order to estimate the attributable deaths, Baccini et al. [25] implemented a PS matching on the same data set used in the present paper, after dichotomizing the exposure according to a threshold of 40 *μ*g/m^3^. In that work, the level of mortality observed in days with exposure values exceeding the threshold was contrasted to the level of mortality we would have observed in those days had the levels of exposure been set to a value below the threshold. Differently, the approach proposed in the present paper does not require the definition of a binary version of the exposure, but allows to treat the air pollutant concentration as a continuous variable. Moreover, the number of attributable deaths is estimated following the shape of the (potentially nonlinear) average exposure-response curve.

In order to quantify the number of attributable events we proposed two estimands, *AD* and *DAD*. Both of them can be used to measure the impact on mortality of hypothetical interventions able to reduce the air pollutant levels (assuming that the effects of these hypothetical interventions occur only through the reduction of the air pollutant levels). Fixing a threshold for the daily exposure, *AD* compares the actual level of mortality with the level of mortality we would observe if the daily exposures exceeding the threshold were exactly equal to the threshold. On the contrary, *DAD* compares the actual level of mortality with the level of mortality we would observe if we were able to intervene on the days with daily exposures exceeding the threshold and set instead the level exposure to a value below the threshold, according to a hypothetical distribution – for example, the observed distribution of the exposures lower than the threshold.

It is worth noting that, while the aDRF averages the potential outcomes over all days in the study period, *AD*(*z*^⋆^) and *DAD*(*z*^⋆^) are sums defined only over the “treated” days, i.e. the days characterized by exposure levels higher than the threshold *z*^⋆^. In this sense, these quantities are somewhat related to the so-called average causal effect on the treated (ATT) [22].

Our analysis found that the estimated aDRFs were not linear. This was true also after a log transformation (not reported), thus indicating that the usual log-linearity assumption was, in this case, not valid. Moreover, being the estimated aDRFs steeper for low values of the exposure, our results seem to indicate that a "safe" threshold does not exist. As a consequence, any reduction of the PM_10_ levels, even when they are already under the actual regulatory limits, is expected to lead to a decrease in the health burden attributable to the exposure. The idea that there is no threshold, acknowledged also by governmental and international agencies [17, 46, 49], is supported by the observation that the population is a mixture of biologically different individuals with various levels of susceptibility, and, as a consequence, no threshold below which nobody experiences the health effect can be detected, especially in the presence of frail (and potentially hypersusceptible) subjects (e.g. children, elderly people, etc.). Further considering the shape of the aDRF, while the presence of a plateau for high concentration values (as for natural-cause mortality) would suggest that small reductions of PM_10_ are ineffective in highly polluted days, the absence of a clear plateau (as for respiratory and cardiovascular mortality) suggests that any measure aimed to produce a reduction (even small) of air pollutant concentration levels can prevent deaths, regardless the initial level of exposure.

We observed evidence of a stronger effect of PM_10_ on natural-cause mortality in days characterized by higher temperature. This finding seems consistent with a previous investigation conducted on the entire territory of the Lombardy region (for which Milan is the capital city), where higher effects of PM10 exposure on mortality were observed during summer [35].

In this paper, aimed at developing a new approach to estimate the dose-response curve and novel estimands for impact assessment, we focused on data on only one city. In the case of studies on multiple locations – which are common in this field – it is possible to use frequentist or Bayesian multivariate meta-analysis methods to combine the derivatives of the estimated aDRFs arising from several locations/cities [50]. We leave this extension to future work.

### Comparison with previous results

The impacts estimated using the proposed approach partly confirmed the results reported in previous papers, obtained on the same data set with different methods. In Table 4 we report:

- the impact of the annual average PM_10_ level exceeding 40 *μ*g/m^3^, calculated using the estimated coefficient from a Poisson regression, thus assuming the log-linearity of the effect [37] (*Regression 40* in Table 4);

**Table 4:** Attributable deaths in Milan, Italy (2003-2006) calculated using different approaches

Source	Natural Deaths		Respiratory Deaths		Cardiovascular Deaths	
	Estimate	90% CI	Estimate	90% CI	Estimate	90% CI
GPS- *AD*(40)	1157	(689, 1645)	312	(210, 418)	771	(580, 961)
GPS- *DAD*(40)	1857	(1479, 2233)	403	(293, 504)	1014	(815, 1221)
*PS matching 40*	1079	(116, 2042)	305	(17, 593)	716	(117, 1315)
*Regression 40*	358	(156, 560)				

GPS- *AD*(40): deaths attributable to daily exposure levels above 40 *μ*g/m^3^, setting the counterfactual exactly to 40 *μ*g/m^3^.

GPS- *DAD*(40)): deaths attributable to daily exposure levels above 40 *μ*g/m^3^, sampling the counterfactuals from the exposure distribution below 40 *μ*g/m^3^.

*PS matching 40*: from Baccini et al. [25], deaths attributable to daily exposure levels above 40 *μ*g/m^3^, calculated according to a PS matching.

*Regression 40*: from Baccini et al. [37], deaths attributable to exceeding the limit of 40 *μ*g/m^3^ for the annual average level of exposure, calculated from a regression approach
- the impact of daily PM_10_ levels above vs. below 40 *μ*g/m^3^, estimated using a PS matching, after dichotomizing the exposure at 40 *μ*g/m^3^ [25]. In this case, mortality in each “treated” day was compared to mortality in a matched day with similar characteristics (similar PS) but exposure under 40 *μ*g/m^3^ (*PS matching 40* in Table 4);
- our current findings *AD*(40) and *DAD*(40).

These estimates deserve some comments. First of all, the number of AD for natural causes estimated through the standard regression approach is far smaller than the other ones reported. This is due in part to the fact that, in the regression approach, the observed mortality was compared to the mortality that we would have observed during the study period, had the annual average level of exposure been set to 40 *μ*g/m^3^^6^. This is different from setting each counterfactual daily level of exposure above 40 *μ*g/m^3^ to exactly 40 *μ*g/m^3^ or to values below 40 *μ*g/m^3^, as in the present paper and in the PS matching approach [25]. In addition, we cannot exclude that the low estimated impacts can partly originate from the incorrect assumption of log-linearity on the exposure-response function.

Secondly, the estimated impacts using the GPS approach, especially *AD*(40), are consistent with the results obtained using PS matching. This is indicative of the substantial reproducibility of the results when applying procedures that, like these, do not force the log-linearity of the effect. At the same time, it also suggests that the simpler PS approach based on a binary version of the exposure can be considered a good "approximation" of the more complex method based on the GPS. However, the GPS approach, which estimates the attributable events according to a curve, returned more precise estimates than those obtained through PS matching in all the investigated outcomes, as shown by the narrower confidence intervals.

Finally, it is worth noticing that the impact *DAD*(40) estimated here is larger than the impact based on PS matching, even if both estimands seem to answer to the same research question: what would be the impact of a hypothetical intervention able to set daily exposure levels under 40 *μ*g/m^3^? The reason of this difference is that, as a matter of facts, the two estimands do not consider exactly the same counterfactual scenario. In fact, according to definition 4, *DAD*(40) and the PS matching estimand share the same threshold *z*^⋆^=40, but set different *p*^⋆^(*z*): for *DAD*(40)*p*^⋆^(*z*) is the observed distribution of the daily exposures for all days with observed levels under the threshold, whereas for PS matching it is the distribution of the daily exposures among the matched controls [25]. Since in PS matching the control days with a level of exposure below 40 *μ*g/m^3^ are matched to the “treated” ones according to similarity in the observed characteristics, it is likely that the matched controls are those with a level of exposure closer to the threshold. For this reason, the impact estimated using PS matching was necessarily lower than *DAD*(40) and close to *AD*(40). This comparison highlights the need of providing a clear definition of the counterfactual scenario that is chosen to assess the impact.

### Study limitations

Our study has some limitations. First of all, for low exposures, when the information is quite poor, a higher degree of smoothing for the bivariate spline of the outcome models would be needed, in order to reduce the sensitivity of the aDRFs to knots location. Adaptive splines could be used to reduce this problem [51, 52]. Second, the bootstrap procedure that we implemented in this analysis assumed independence between units. More complex approaches which account for the auto-correlation in the mortality time series could be used, such as block bootstrap or residual bootstrap methods [53]. Third, checking the balancing property of the GPS is crucial and not straightforward; we implemented a GPS blocking-based approach which employed *t* statistics to quantify unbalance [26]. As an alternative, standardized differences could be used as recently proposed [54].

It also seems appropriate to make a consideration regarding the positivity assumption. In this context, the positivity assumption would state that, for all possible strata defined by the covariates, it is possible to observe any level of exposure. As a matter of fact, even if in principle our approach does not require the validity of this assumption, its strong violation could challenge estimation, due to the presence of regions of the confounder space where inference would rely on extrapolation. A widely used method to address the violation of the positivity assumption consists in trimming the sample, which means excluding from the analysis the classes of units with limited variability in *Z*_*i*_ [55]. Although a slight evidence of violation of the positivity assumption arose in our data, we decided to perform the analysis on the entire data set anyway, without any kind of trimming. This choice was motivated by the need of quantifying the overall burden of mortality attributable to air pollution during the study period, without excluding any “treated” day.

Finally, formal statistical tests on the shape of the aDRF have not been developed within the GPS approach. As a consequence, the non-linearity of the aDRF could not be formally tested and our results on effect modification by temperature, although suggestive of a larger effect during the warm season, should be interpreted in a descriptive sense.

## Conclusion

We adopted a method based on the GPS to obtain a semi-parametric estimate of the average dose-response function describing the short-term effect of airborne particles on mortality and we defined novel estimands to assess the impact in terms of attributable deaths, according to the estimated curve. The estimands allow the specification of different counterfactual scenarios defined on the distribution of the daily air pollutant levels. This approach can be easily extended to other environmental epidemiology contexts. Its potential as a tool to investigate effect modification by temperature or other relevant factors deserves investigation.

## Data Availability

The datasets analysed during the current study and the code are available from the corresponding author on reasonable request.

## Abbreviations

aDRF: average Dose-Response Function
AD: Attributable deaths
CI: Confidence interval
DAD: Distributional Attributable Deaths
GPS: Generalized Propensity Score
PM10: Particulate matter ≤10*μ*m in diameter
PS: Propensity Score
SUTVA: Stable Unit Treatment Value Assumption.

## Footnotes

1 The weak unconfoundedness assumption states the conditional independence between exposure and potential outcomes: $Y_{i}(z) \perp \!\!\!\perp Z_{i} \mid \mathbf {X}_{i} \quad \forall z\in \mathcal {Z}, \forall i$.

2 Unconfoundedness given the GPS: $Y_{i}(z) \perp \!\!\!\perp Z_{i} \mid r(z; \mathbf {X}_{i})\quad \forall z\in \mathcal {Z}, \forall i$

3 It is worth noticing that, for a fixed value of the GPS, the regression function of Y given the exposure “does not have a causal interpretation” [26]. This means that the regression model does not directly result in the causal aDRF, which instead must be derived using the procedure described in the “Estimation procedure” section

4 $\widehat {R}_{i}=\frac {1}{\sqrt {2\pi \hat {\sigma }_{Z}^{2}}}\exp {-\frac {\left (\log (Z_{i})-\hat {\alpha }_{0}-\hat {\vec \alpha }_{X}^{T}\mathbf {X}_{i}\right)^{2}}{2\hat {\sigma }_{Z}^{2}}}$

5 $\hat {r}(z; \mathbf {X}_{i})=\frac {1}{\sqrt {2\pi \hat {\sigma }_{Z}^{2}}}\exp {-\frac {\left (\log (z)-\hat {\alpha }_{0}-\hat {\vec \alpha }_{X}^{T}\mathbf {X}_{i}\right)^{2}}{2\hat {\sigma }_{Z}^{2}}}$

6 The number of attributable deaths due exceeding the threshold *T* for the annual average level of the air pollutant was calculated as *y*−*y*/*e**x**p*(*b*(*x*−*T*))*I*(*x*>*T*),where *y* is the observed number of deaths during the study period, *x* is the annual average level of the air pollutant, *b* is the estimated regression coefficient expressing the air pollutant effect and *I*(*x*>*T*) is an indicator function which is equal to 1 if *x*>*T* and 0 elsewhere [37].
